# Remarks on Sinus Phlebitis and Thrombosis Complicating Suppurative Middle Ear Disease

**Published:** 1921-09

**Authors:** J. P. I. Harty

**Affiliations:** Hon. Surgeon in charge of Ear, Nose and Throat Department, Bristol Royal Infirmary


### The Bristol Medico-Chirurgical Journal.

*" Scire est nescire, nisi id me*
*Scire alius sciret."*

SEPTEMBER, 1921.

### REMARKS ON SINUS PHLEBITIS AND THROMBOSIS COMPLICATING SUPPURATIVE MIDDLE EAR DISEASE.

(*With notes of nine illustrative cases.*)

BY

J. P. I. HARTY, B.A., M.B., B.Ch. (R.U.I.)., F.R.C.S.,

*Hon. Surgeon in charge of Ear, Nose and Throat Department,*
*Bristol Royal Infirmary.*

INFECTION of the venous sinuses is one of the commonest of the intracranial complications of suppurative middle ear disease. Most frequently it is associated with the chronic form of suppurative otitis media, but it also occurs in the acute form, especially in young subjects.

The tendency from reading text books is to regard inflammation of the mastoid bone as an entity: this is erroneous, as it conforms to inflammation of bone in other regions. In suppurative otitis media we really have a muco-periostitis, markedly so when the mastoid antrum, aditus and cells are involved, which, though not universal, is a very frequent occurrence. Once the inflammation has penetrated beyond the muco-periosteal limits an acute osteomyelitis occurs, and to this condition the name of mastoiditis is usually applied. The terminology of acute and chronic mastoiditis is very confusing, as osteomyelitis may occur, either with an acute or a chronic suppurative otitis media, and especially so as the osteomyelitis does not necessarily involve the mastoid process alone, but may attack other portions of the petro-mastoid, either in combination with or apart from the mastoid process.

Infection of the venous system is very prone to occur in acute inflammation of bone, owing to the large size of the venous radicles and the practical absence of capillaries, while this liability is further increased in the petro-mastoid owing to the very short course of these radicles before entering a large venous sinus. An important point to remember is that while otitis media—the determining factor of the mastoiditis—may subside, the mastoiditis may progress. So suppuration in the mastoid process, with its accompanying complications, can exist with an intact tympanic membrane and no otorrhœa. (*Vide* Cases 4 and 5, but in the latter otorrhœa was present.)

The sigmoid portion of the lateral sinus is usually infected, but phlebitis of the jugular bulb occurs in acute suppurative otitis media, especially in the young. Too much attention is directed to the sigmoid sinus owing to its large size and its proximity to the mastoid cells, and this, in my experience, is a source of error. A healthy sigmoid sinus may lie above a phlebitis, or even thrombosis of the jugular bulb. Aspiration with a hypodermic syringe then only further deludes, and at the same time is always a risky procedure, as cases of infection from wounds of the sinus undoubtedly occur. The fact that general systemic infection can occur through a phlebitis or thrombosis of one or more of the mastoid venules, apart from sinus infection, is a point to be remembered. This explains the subsidence of symptoms which occurs after operation in those cases where the mastoid cells and antrum have been opened, and the sinus exposed, without even the definite exhibition of pus. For these reasons it is my practice, after concluding that the exposed sinus is healthy, to pack the cavity, leaving the postaural wound open in its full extent, and await results. If the temperature and symptoms of systemic infection do not subside, I immediately tie the internal jugular vein and follow on with opening the sinus.

### ROUTES OF INFECTION.

1. The most usual is by direct extension, and this is favoured by a pneumatic or diploëtic mastoid process, or by a combination of these two types. During operation on these cases the sinus is exposed, after the removal of a few flakes of bone, and is very often surrounded by a perisinus abscess. (*Vide* Cases 2, 4, 7, 8, 9.)

2. Through the venous radicles from the mastoid cells opening into the sigmoid sinus. It is in this type that intervening bone, of a more or less healthy appearance and varying thickness, is encountered between the pus-containing mastoid cells and the sinus. It is my experience that a smooth layer of ivory-like bone overlying a sinus always indicates a perisinus abscess with a thrombosed sinus. (*Vide* Cases 3 and 4.)

3. From the superior petrosal sinus. No instance of this is recorded in these notes.

4. Through the floor of the tympanum to the jugular bulb, especially in acute suppurative otitis media and in children, where a dehiscence of the bone may be present. (*Vide* Case 1.)

Other paths of infection do occur *via* the emissary veins, or phlebitis of the jugular bulb and upper part of the internal jugular vein through cellulitis of the neck or scalp, but it is with cases complicating otitis media that this paper deals.

Very often a perisinus abscess is present, or there may be a perisinusitis in which the outer wall is covered with granulations. If thrombosis has taken place the clot may be complete, occluding the lumen of the sinus, or incomplete "mural thrombosis." The clot, once formed, may break down and a sinus abscess result (see Case 3). The actual demonstration of a thrombus is hailed with glee both by the surgeon and onlookers. The curtain rises in the final act; disclosure and ejection of the "villainous clot" is followed by the introduction of clean packing. The scene closes, and everyone returns home satisfied. Thrombosis is Nature's attempt to secure obliteration of the sinus and cut off infection from the blood stream, but it does not occur in every instance. Unrecognisable mural infection of both the sinus and internal jugular vein may extend for quite a considerable distance without any actual thrombosis, as illustrated in Case 8.

Assuming that the sigmoid sinus is the portion infected. The clot, once formed, may extend in various directions:—

1. Backwards to the torcular. It is rare for this to extend to the opposite lateral sinus, but it may proceed along the superior longitudinal or straight sinuses. No instance of this, however, occurred in my series of cases.

2. Along the superior or inferior petrosal sinuses with resulting thrombosis of the cavernous sinus. (*Vide* Case 6.)

3. Downwards to the jugular bulb and along the internal jugular vein. (*Vide* Case 7.)

### SYMPTOMS AND CLINICAL SIGNS.

1. *High Temperature*, 102°–106°, in a case of suppurative otitis media, either acute or chronic, is always suggestive of sinus implication. If there is any history of a definite rigor it is, in a large percentage of cases, pathognomonic. In typical cases the temperature falls to normal, accompanied by profuse perspiration, and two or three of such rigors may occur in severe infections within the 24 hours, while in a fair number of cases rigors may be so slight as to have been overlooked. The pulse-rate is proportionately accelerated to the temperature unless further intracranial complications exist.

2. *Otorrhœa of either the acute or chronic variety.*—If this is absent, the history of a discharge which has lately subsided, or a recent ear-ache, should be elicited.

3. *Deep-seated pain in the mastoid region.*

4. *Mastoid tenderness* is usually, but not invariably, present, and may be associated with overlying œdema, which is particularly marked when thrombosis has extended to the mastoid emissary vein. (*Vide* Case 2.)

5. *The mental condition* is not altered in the uncomplicated cases.

From analysis of my cases the presence of these five is sufficient to justify a diagnosis, but the following cannot be regarded as cardinal :—

6. Tenderness over the course of the upper part of the internal jugular vein and associated swelling of this region of the neck may be present if phlebitis or thrombosis have extended to the vein, and therefore is rather a late symptom, and should in no way be regarded as pathognomonic. Torticollis in association with this has been observed.

7. Optic neuritis is present in a small percentage of cases.

8. Vomiting may occur.

9. The 9th, 10th and 11th cranial nerves may be implicated from thrombosis of the jugular bulb, and the 12th from thrombosis of the anterior condylar vein; but these are rare, and have not occurred in any of my cases.

Pyosepticæmia determines the formation of pulmonary infarcts progressing to abscesses of the lungs, and secondary abscesses from systemic emboli may occur later.

### PROGNOSIS.

*Too much stress cannot be laid upon an early diagnosis.* If active surgical intervention is employed in the early stages of uncomplicated cases, the prognosis is good provided they are seen either before or immediately after general systemic infection has taken place.

### TREATMENT.

The treatment is operative, and involves thorough exposure of the focus of infection, which means either the complete (radical mastoid) operation or the incomplete (Schwartze) according to the condition of the case—and in the majority of instances ligation of the internal jugular vein in the neck. It is my practice to do a lumbar puncture before starting the operation, as the information gained is valuable, and I have never observed any ill results from this procedure.

**Case 1.**—M.S., Bristol Royal Infirmary.

*History.*—Admitted March 9th, 1919, "cold" 7 days previous.

March 8th.—Right ear-ache, followed by discharge. 12th.—Temperature again rising after having reached normal. Apical mastoid tenderness. No bulging of membrane. Antero-inferior perforation with pulsatile discharge. No ear-ache. Swab of meatal discharge, streptococci and diphtheroid organisms.

*Operation* on the 17th.—Schwartze performed, pus found after removing cortical flake of bone. Antrum opened. Sigmoid sinus exposed but apparently healthy. Meatal discharge rapidly ceased but temperature remained high.

20th.—Lumbar puncture, fluid clear, no pressure. Culture sterile. 21st.—Rapid rise of temperature in evening. 22nd.—Optic discs examined (Dr. Stack), normal.

24th and 25th.—Rapid rises of temperature falling to normal. No definite rigor. No pain. No tenderness over internal jugular vein. Mastoid wound clean, no meatal discharge.

Sample of blood taken for culture. Pathological report: streptococci.

26th. *Second Operation.*—Ligature of the internal jugular vein above and below the common facial vein with excision of the intervening portion and division after ligature of the common facial vein. Opening of the sigmoid sinus after occlusion of the torcular end by extradural packing. Sinus exposed well down towards the bulb and the outer wall of sinus removed. Clot found practically in bulb.

29th.—Temperature falling, tender swelling right thigh, just below and behind anterior superior iliac spine. 30th.—Temperature again rising. 31st.—Swelling and tenderness of thigh disappearing.

April 3rd.—Abscess in neck (suppuration from lower end of upper portion of divided internal jugular vein) opened by removal of a few stitches. Uneventful recovery with healing of neck and post-aural wounds. May 19th.—Perforation of tympanic membrane healed. Hearing of right ear practically normal. Whispered words heard at 30 feet.

**Case 2.**—Boy, aged 8 years. May 30th.—Tonsils and adenoids operation. June 7th.—Admitted in evening. Discharge from right ear (acute suppurative otitis media June 2nd, vomited on evening of June 6th). Temperature 102°. Deep fluctuating swelling beneath right sternomastoid extending backwards to sub-occipital region, and marked mastoid œdema. Von Bezold's mastoiditis diagnosed.

*Operation* (9.15 p.m.).—Lumbar puncture—fluid clear Mastoid abscess, which had burst through inner surface of mastoid process communicating with abscess beneath sterno mastoid. Large perisinus abscess, with thrombosis of mastoid emissary vein and pus passing out through mastoid foramen beneath pericranium. Thrombosis of sigmoid sinus. Ligation of internal jugular vein in neck and lower end of upper portion brought out in neck.

Sinus thoroughly exposed and clot cleared out. Free bleeding from upper end. Plugged and outer wall of sinus removed.

Subsequently complained of severe pain in both legs. Temperature continued to oscillate.

June 14th.—Lumbar puncture. No meningitis. This was done as it was thought that the first puncture might have determined a spinal meningitis which would account for the pain in the legs.

June 16th.—Died. Post-mortem: death from septicæmia. No meningitis spinal or cranial. Thrombosis of right middle cerebral artery (congenital syphilis ?).

**Case 3.**—Male, aged 28. June 26th, 1920. Admitted in evening. Discharge from right ear for two years. Severe headaches for four days. History of two rigors previous evening and following morning. Temperature 103.4°. Tenderness and œdema over mastoid. Retraction of head. Semi-comatose.

*Immediate operation.*—Lumbar puncture—definite meningitis. Pus in mastoid. Sinus exposed. Ligature of internal jugular vein. On opening sinus pus was found and this communicated with subarachnoid space. Cerebro-spinal fluid and pus welled out. A coarse nystagmus on looking to right developed. Optic discs slightly pale, otherwise normal (Dr. Stack).

July 3rd.—Patient gradually collapsed and died. Post-mortem not granted.

**Case 4.**—Male, aged 16. Admitted April 15th, 1920. Previous history right otorrhœa, none now. Right ear-ache started a week ago. Pain in head and ear. No discharge from right ear. Oedema behind ear. Temperature 103.°

*Immediate operation.*—Lumbar puncture, no meningitis. Pus in mastoid. Thrombosed lateral sinuses opened and clot cleared out after ligature of internal jugular vein in neck.

Uneventful convalescence. Double optic neuritis about 3 D. of swelling (Dr. Stack). Organisms from mastoid swab, bacillus coli com. and staphylococci.

**Case 5.**—A. E. Y., male, aged 45. June 7th, 1920. Attended as out-patient. Right otorrhœa, deafness and tinnitus. No perforation of membrana tympani to be made out, but what was first thought to be deep furuncle on posterior meatal wall discharging pus. This was a sinus leading to mastoid antrum, the otitis media having subsided. Attending as out-patient daily.

July 6th.—Rigor in morning. Admitted in evening. Pain and tenderness over right mastoid. No œdema. Rigor with temperature 103° just after admission.

*Immediate operation.*—Pus in mastoid. Thrombosed lateral sinus. Clot cleared out, ligature of internal jugular vein, etc. Subsequent stitching of post-aural wound with formation of meatal flaps and skin grafting. Membrana tympani intact. Wound in neck healed. Hearing whispered words 6 feet.

**Case 6.**—E. B., male, aged 3 months. Admitted June 20th, 1919. Swelling behind left ear. Acute suppurative otitis media right and left. History two to three days.

*Operation.*—June 23rd. Large left subperiosteal abscess opened and antrum exposed. Oedema of right face with proptosis and symptoms of meningitis (seen by Dr. Stack), this had developed during the night. Died.

*Post-mortem.*—Necrosis of practically the whole of right petrous bone, thrombosis of lateral, superior and inferior petrosal and cavernous sinuses on the right side, meningitis. The left side, which had been operated on, was apparently in a healthy condition.

**Case 7.**—Pte. J. C., aged 22. Admitted July 6th, 1918. Chronic suppurative otitis media right for years. Polypus filling meatal depths. Temperature 103°, some pain right ear. No definite mastoid or internal jugular vein tenderness. No rigors. (Influenza epidemic at the time.)

Polypus removed, local anæsthesia.

July 8th.—Some œdema of postero-superior meatal wall.

*Operation.*—Lumbar puncture. Fluid clear. Pus in antrum, extradural abscess. Large perisinus abscess with thrombosis of sigmoid sinus. Ligature of internal jugular vein, which was thrombosed.

Died July 9th.

*Post-mortem.*—Pyæmia. Abscesses of both lungs. Slight meningitis.

**Case 8.**—M. S., female, aged 19. Admitted July 24th, 1920. Four months' history right otorrhœa. Pain followed by swelling behind ear started five days ago.

*Operation.*—Pus in antrum. Extradural abscess. Lateral sinus exposed, appeared normal.

July 26th.—Temperature 103°. Fundi oculi normal (Dr. Stack). July 28th.—Temperature 105°. Dressed. Suspicious patch on lateral sinus and some doubtful tenderness over internal jugular vein.

*Operation.*—Ligation, etc., of internal jugular vein. On removal of post-aural packing the suspicious patch on the sinus gave way with profuse hemorrhage. The sinus was opened, etc. Temperature gradually fell to normal, and remained until August 4th, when a rigor (temperature 105°) occurred at midnight. August 5th another rigor in the morning. Taken to theatre. Neck wound opened up and lower portion of divided internal jugular vein found infected. Removal as low down as possible.

Temperature sank to normal. Uneventful recovery.

**Case 9.**—A. P., female, aged 30. Admitted March 5th, 1921.

*History.*—Discharge from both ears since childhood. Rigor, pain and tenderness over left mastoid. Tenderness over left internal jugular vein.

*Operation.*—Lumbar puncture, fluid clear. Sclerosed type of mastoid. Pus in antrum. Lateral sinus found thrombosed. Ligature, etc., of internal jugular vein. Sinus opened and clot removed, etc.

March 14th.—Convalescence was interrupted by a three months' abortion, when there was severe hemorrhage, and the uterus had to be cleared out under a general anæsthetic.

The percentage mortality as shown in this series of cases is naturally too high, as they are chosen for illustrative purposes. Taking all the operations I have performed, the death-rate is just over 30 per cent. Case 1 is interesting from the fact that, though septicæmia (blood culture) was present before operation, recovery, with practically normal hearing, resulted.

In conclusion, I must thank Dr. P. Watson-Williams both for his assistance and for permission to publish this series, as the great majority of the cases occurred while he was in charge of the Ear, Nose and Throat Department of the Bristol Royal Infirmary.

